# Nipple sparing mastectomy in breast cancer patients and long-term survival outcomes: An analysis of the SEER database

**DOI:** 10.1371/journal.pone.0183448

**Published:** 2017-08-25

**Authors:** Mingzhu Li, Kai Chen, Fengtao Liu, Fengxi Su, Shunrong Li, Liling Zhu

**Affiliations:** 1 Quanzhou First Hospital Affiliated to Fujian Medical University, Quanzhou, Fujian, China; 2 Guangdong Provincial Key Laboratory of Malignant Tumor Epigenetics and Gene Regulation, Sun Yat-Sen Memorial Hospital, Sun Yat-Sen University, Guangzhou, Guangdong, China; 3 Breast Tumor Center, Sun Yat-sen Memorial Hospital, Sun Yat-sen University, Guangzhou, Guangdong, China; 4 Department of Biostatistics, Yale School of Public Health, Yale University, New Haven, Connecticut, United States of America; University of North Carolina at Chapel Hill School of Medicine, UNITED STATES

## Abstract

**Purpose:**

To determine the prevalence of nipple-sparing mastectomy (NSM) and its long-term survival outcomes in breast cancer patients.

**Method:**

We used the Surveillance, Epidemiology, and End Results database and identified 2,440 breast cancer patients who received NSM during 1998–2013. We used chi-square and binary logistic regression to identify factors associated with the use of radiotherapy after NSM. We used Kaplan-Meier analysis to estimate cancer-specific survival (CSS) and overall survival (OS). We used the log-rank test and Cox regression to identify factors associated with CSS and OS.

**Results:**

The median age of the population was 50 years. There were 725 (29.7%), 1064 (43.6%) and 651 (26.7%) patients who had Tis, T1 and T2-3 disease and 1943 (79.6%), 401 (16.4%) and 96 (3.9%) patients who had N0, N1 and N2-3 disease, respectively. The rates of RT use were 61.4%, 39.6% and 10.9% in patients with N2-3 disease, N1 or T3/N0 disease and Tis/T1-2N0 disease, respectively. Elderly age, African American race, and higher T-stage and N-stage were associated with receiving radiotherapy. For patients diagnosed between 1998–2010 (N = 763), the median follow-up was 69 months. The 5- and 10-yr CSS were 96.9% and 94.9%, respectively. The 5- and 10-yr OS were 94.1% and 88.0%, respectively. Ethnicity, T-stage and N-stage were factors independently associated with CSS, and age and T-stage were factors independently associated with OS.

**Conclusions:**

The use of NSM has increased, and it is oncologically safe for breast cancer patients.

## Introduction

Nipple-sparing mastectomy (NSM) is a procedure that aims to preserve the nipple areolar complex and skin when performing a mastectomy to achieve better aesthetic outcomes after breast reconstruction. It is a popular surgical approach for patients receiving prophylactic mastectomy. Several studies[1–6] have reported the use of NSM as a therapeutic approach for breast cancer patients and its oncological outcomes. However, most of these studies were based on patients in a single institution and thus lacked sufficient generalizability. The National Comprehensive Cancer Network (NCCN) guidelines[7] state that NSM could be a surgical option in highly selected patients (e.g., those with a favorable prognosis)[7] and acknowledged that only retrospective studies were available as evidence. The nationwide patterns of NSM utilization in breast cancer patients and its associated long-term clinical outcomes remain unknown. The Surveillance, Epidemiology, and End Results (SEER) database is a national database covering one third of the cancer patients in the US. In this study, we aimed to use the SEER database to study 1) trends in the use of NSM between 1998 and 2013, 2) the long-term cancer-specific survival (CSS) and 3) the utilization of radiotherapy in this populations.

## Methods

We searched the SEER registry data from 18 registries (Nov. 2015 submission) and included breast cancer patients who met all of the following inclusion criteria (S1 File).

### Inclusion criteria

1. Female patients with pathological diagnosis of malignant disease of the breast. Carcinoma in situ patients were also included.
2. Patients who underwent nipple sparing mastectomy (code 30 in the SEER database)[8].
3. Patients diagnosed between 1990 and 2012.
4. Tis and T1-3 patients.

### Exclusion criteria

1. Bilateral breast cancer patients.
2. Patients with previous diagnosis of any malignant tumors.
3. Patients with a phyllodes tumor of the breast.
4. Patients with inconsistent coding identified within the SEER database. For example, patients coded as carcinoma in situ and T2 or T3 were considered to have inconsistent coding.
5. Patients with unknown follow-up status.

This was an epidemiological study using de-identified data from the SEER registry. Ethical approval by the ethical committee of the Quanzhou First Hospital and Sun Yat-sen Memorial Hospital was waived based on our institutional policy. This study was reported based on the REMARK statements[9].

The following data were extracted for each patient: age, race, year of diagnosis, county type, marital status at diagnosis, grade, adjusted AJCC 6th T-stage, tumor size, primary site, histology subtype, estrogen receptor (ER) status, progesterone receptor (PR) status, radiation therapy (RT) use, survival month and SEER cause-specific death classification. Patients were categorized into three age groups based on their age at diagnosis (<50 yrs, 50–69 yrs, ≥70 yrs). Histology was divided into four categories (carcinoma in situ, infiltrating ductal carcinoma, lobular carcinoma and others).

### Statistical analysis

A descriptive analysis of the patient characteristics was conducted. For survival analysis, the median follow-up was calculated as the median observed survival time of the entire population. To ensure the length of the follow-up to be sufficient, only patients from 1998–2010 were included in the survival analysis. Cancer-specific survival (CSS) and overall survival (OS) were measured as the time from diagnosis to breast cancer-related death and death due to any reasons, respectively. Cumulative CSS and OS were estimated using Kaplan-Meier analyses. We used the log-rank test to identify univariate risk factors for CSS and OS. We used the Cox proportional hazards regression to confirm risk factors independently associated with CSS and OS. We used Schoenfeld residuals to test the proportional-hazards assumptions. All *P*-values were two-sided. *P*-values of less than 0.05 were as considered statistically significant. Data were obtained using SEER*STAT 8.2.1. All data will be available upon request with a signed contract for data transfer agreement with the authors. The statistical analysis was performed using Stata/MP, version 13.0 (StataCorp LP, College Station, TX, USA) and R.

## Results

### Clinicopathological features

**Fig 1:**
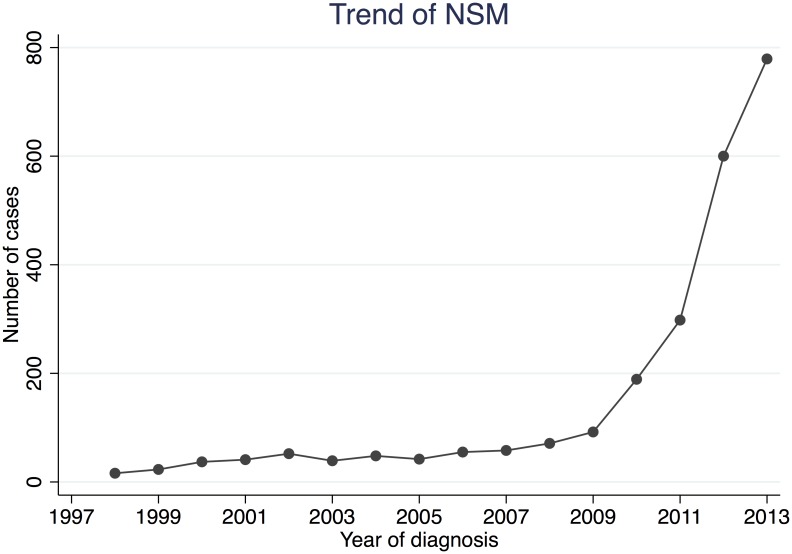
The use of NSM recorded in the SEER database during the study period (1998–2013).

A total of 2,440 patients were included in this study (Table 1). Before 2009, less than 100 cases/year were coded as NSM in the SEER database. After 2009, the number of NSM increased rapidly, reaching 779 in 2013 (Fig 1). The median age of the entire population was 50 years. In total, 725 (29.7%), 1,064 (43.6%), 568 (23.3%) and 83 (3.4%) patients had Tis, T1, T2 and T3 disease, respectively. There were 1,943 (79.6%) patients with negative axillary nodes. There were 417 (17.1%) and 1,933 (79.2%) patients who did and did not receive RT, respectively. We also looked at the indications for RT in this cohort. Based on the indications for post-mastectomy RT provided by the NCCN guidelines, we categorized the patients into “RT recommended”, “RT considered” and “RT not recommended” cohorts, which consisted of patients with N2-3 disease, N1 or T3/N0 disease, and Tis/T1-2N0 disease, respectively (Table 2). In total, RT rates were 61.4%, 39.6% and 10.9% in the “RT recommended”, “RT considered” and “RT not recommended” cohorts, respectively. Over the entire study period, the RT rate fluctuated between 10–20% in the “RT not recommended” cohort. In the “RT considered” cohort, the rate of RT increased steadily between 2005 and 2013. In contrast, the rate of RT in the “RT recommended” cohort decreased from 80% during the 2001–2004 period to 60% during the 2001–2013 period.

**Table 1:** Clinicopathologial features of included patients.

	N	%
**Age Group**		
<50	1,165	47.75
50–69	1,135	46.52
> = 70	140	5.74
**Year Of Diagnosis**		
1998–2002	169	6.93
2003–2007	242	9.92
2008–2013	2,029	83.16
**County Type 2003**		
Metropolitan	2,253	92.34
Non-Metropolitan	184	7.54
Unknown	3	0.12
**Race**		
White	1,865	76.43
African American	277	11.35
Others	275	11.27
Unknown	23	0.94
**Marital Status**		
Married	1,595	65.37
Divorced/Separated/Single/Widowed	770	31.56
Unknown	75	3.07
**Primary Site**		
Nipple/Central Portion	113	4.63
UIQ	251	10.29
LIQ	140	5.74
UOQ	742	30.41
LOQ	226	9.26
Overlapping/Unknown	500	20.49
NOS	468	19.18
**T-Stage**		
Tis	725	29.71
T1	1,064	43.61
T2	568	23.28
T3	83	3.40
**N-Stage**		
N0	1,943	79.63
N1	401	16.43
N2	69	2.83
N3	27	1.11
**Histology**		
In situ	725	29.71
IDC	1,227	50.29
ILC	199	8.16
Others	289	11.84
**Grade**		
I	415	17.01
II	998	40.90
III	766	31.39
IV	60	2.46
Unknown	201	8.24
**ER**		
Negative	336	13.77
Positive	1,862	76.31
Unknown	242	9.92
**PR**		
Negative	519	21.27
Positive	1,635	67.01
Unknown	286	11.72
**Radiation Therapy**		
No	1,933	79.22
Yes	417	17.09
Unknown	90	3.69

ER, estrogen receptor; PR, progesterone receptor; LIQ, lower-inner quadrant; LOQ, lower-outer quadrant; UIQ, Upper-inner quadrant; UOQ, Upper-outer quadrant; NOS, Non otherwise specified.IDC, infiltrating ductal carcinoma; ILC, Invasive lobular carcinoma.

**Table 2:** Temporal trends in the use of RT after NSM by NCCN recommendations.

Year	RT not recommended		RT considered		RT recommended	
	%	Number of RT/ Total number	%	Number of RT/ Total number	%	Number of RT/ Total number
1998–2000	23.73%	(14/59)	28.57%	(2/7)	37.50%	(3/8)
2001–2004	15.60%	(22/141)	25.81%	(8/31)	80.00%	(4/5)
2005–2007	9.45%	(12/127)	27.27%	(6/22)	75.00%	(3/4)
2008–2010	18.56%	(49/264)	38.98%	(23/59)	66.67%	(10/15)
2011–2013	8.24%	(104/1262)	42.41%	(123/290)	60.71%	(34/56)

RT, Radiotherapy

### Survival analysis

For the survival analyses, we included patients diagnosed between 1998–2010 as the study cohort (N = 763). With a median follow-up of 69 months, the 5- and 10-yr CSS rates were 96.9% and 94.9%, respectively. The 5- and 10-yr OS rates were 94.1% and 88.0%, respectively. Estimations of CSS and OS stratified by patient and tumor characteristics are summarized in Table 3. Univariate analysis using the log-rank test showed that race (P = 0.003), T-stage (P<0.001), N-stage (P<0.001), histology (P<0.001) and RT status (P<0.001) were significantly associated with CSS. Multivariate analysis (Table 4) showed that African American race (vs. White/others/Unknown, HR = 3.21, 95% CI 1.39–7.41) and higher T-stage (T1 vs. Tis, HR = 8.59, 95% CI 1.06–69.34; T2-3 vs. Tis, HR = 18.52, 95%CI 2.25–152.75) and N-stage (N1-3 vs. N0, HR = 2.41, 95%CI 1.06–5.48) were independent risk factors for CSS. For OS, age (P<0.001), race (P = 0.010), marital status (P = 0.002), T-stage (P<0.001), N-stage (P<0.001), histology (P = 0.005) and RT status (P = 0.013) were significantly associated with OS. Multivariate analysis suggested that age (≥70yrs. Vs. <50yrs: HR = 4.81, 95%CI 2.46–9.40) and T-stage (T2-3 vs. Tis, HR = 2.78, 95%CI 1.32–5.84) were independent risk factors for OS. RT was not significant as an independent risk factor for CSS and OS.

**Table 3:** 5-year and 10-year cumulative CSS and OS of patients with different clinicopathological features.

	Cancer-specific survival			Overall survival		
	5-year	10-year	Log-rank Test, P	5-year	10-year	Log-rank Test, P
Age group						
<50	95.97	93.01	NS	95.4	90.8	<0.001
50–69	98.13	96.47		95.69	88.86	
> = 70	95.69	95.69		78.87	69.5	
County type 2003*						
Metropolitan	96.89	95.46	NS	94.24	88.65	NS
Non-metropolitan	96.97	90.93		93.1	82.86	
Race*						
White	97.63	95.18	0.003	95.08	88.38	0.01
African American	88.54	88.54		82.94	79.48	
Others	100	100		98.48	94.55	
Marital status*						
Married	97.46	94.92	NS	96.03	91.23	0.002
Divorced	95.66	94.33		90.35	82.33	
Primary Site						
Nipple/Central portion	100	88.89	NS	94.59	84.08	NS
UIQ	98.57	95.39		97.14	94.01	
LIQ	94.5	94.5		94.5	94.5	
UOQ	97.24	94.46		94.96	88.62	
LOQ	100	94.44		91.52	86.43	
OVERLAPPING/UNKNOWN	95.18	95.18		92.67	86.47	
NOS	96.62	96.62		93.82	87.42	
T-stage						
Tis	99.6	99.6	<0.001	97.9	93.1	<0.001
T1	97.81	94.95		96.11	89.6	
T2	90.49	85.18		84.92	75.87	
T3	91.64	83.31		84.28	76.62	
N-stage						
N0	98.63	97.44	<0.001	95.68	90.98	<0.001
N1	89.76	81.28		88.96	72.71	
N2	90.34	90.34		86.04	76.48	
N3	75	75		68.18	68.18	
Histology						
In situ carcinoma	99.6	99.6	<0.001	97.9	93.1	0.005
IDC	93.94	91.26		91.51	87.76	
ILC	97.5	82.03		95.73	69.73	
Others	97.88	95.93		90.64	82.23	
Grade						
I	100	98.36	NS	95.61	89.96	NS
II	97.24	93.66		94.9	86.44	
III	93.99	92.67		92.17	88.75	
IV	97.06	97.06		92.14	83.32	
Unknown	97.94	96.65		94.78	90.57	
ER						
Negative	92.39	92.39	NS	90.14	90.14	NS
Positive	97.29	94.5		94.57	88.24	
Unknown	97.7	96.64		94.3	87.58	
PR						
Negative	94.27	93	NS	93.54	86.26	NS
Positive	97.02	94.17		93.58	89.11	
Unknown	97.95	96.91		94.9	88.32	
Radiation therapy						
No	97.79	96.2	<0.001	94.87	89.62	0.013
Yes	95.49	92.95		92.85	83.87	
Unknown	82.25	65.8		82.25	65.8	

ER, estrogen receptor; PR, progesterone receptor; LIQ, lower-inner quadrant; LOQ, lower-outer quadrant; UIQ, Upper-inner quadrant; UOQ, Upper-outer quadrant; NOS, Non otherwise specified.IDC, infiltrating ductal carcinoma; ILC, Invasive lobular carcinoma. NS, Non-significant; CSS, Cancer-specific survival; OS, Overall survival;

* Patients with unknown status were excluded. Separated/Single/Widowed were included in the divorce category.

**Table 4:** Independent risk factors for CSS and OS in Cox proportional hazard regression analysis.

	Cancer specific Survival		Overall Survival	
	HR(95%CI)	P	HR(95%CI)	P
Age				
<50	Not included		1	
50–69			1.37(0.74–2.44)	0.325
> = 70			4.81(2.46–9.40)	<0.001
Marital*				
Married	Not included		1	
Divorced			1.55(0.92–2.61)	0.099
Race				
White/Others/Unknown	1.00		1	
African American	3.21(1.39–7.41)	0.006	0.58(0.31–1.09)	0.091
T-stage				
Tis	1.00		1	
T1	8.59(1.06–69.34)	0.044	1.18(0.58–2.39)	0.648
T2-3	18.52(2.25–152.75)	0.007	2.78(1.32–5.84)	0.007
N-stage				
N0	1.00		1	
N1-3	2.41(1.06–5.48)	0.036	1.57(0.85–2.89)	0.149
Radiotherapy				
No	1.00		1	
Yes	1.02(0.43–2.42)	0.962	0.96(0.53–1.72)	0.894
Unknown	3.86(1.24–12.01)	0.020	2.26(0.79–6.43)	0.126

Significant factors associated with CSS shown in univariate analysis (race, T-stage, N-stage and RT) were included in the Cox regression. Histology was excluded due to collinearity with T-stage. Univariate analysis suggested that African American patients had significantly lower CSS and OS than the other categories. Thus, we combined the "White", "Others" and "Unknown" categories as one category.

* Separated/Single/Widowed were included in the divorce category.

## Discussion

The registries of the SEER Program routinely collect data on patient demographic characteristics, clinicopathological features and vital status follow-up. The SEER database therefore serves as a reliable source of information for the investigation of the prevalence of and clinical outcomes associated with NSM. NSM performed was increased significantly after 2009. It is possible that patients receiving NSM were not appropriately coded as having undergone a NSM before 2009. Addtionally, NSM was first used in the prophylactic settings, and therefore the numbers may actually be higher. Its use in breast cancer patients should be cautious and based on its oncological safety indicated by the published studies. We searched the Pubmed using the terms “Nipple sparing mastectomy breast cancer” in all fields and we observed that the number of identified publications had not been significantly increased until 2007-2009(S1 Fig). This data suggested that the oncology community began to accept and embrace the NSM in breast cancer treatment after 2009.

Agarwal et al.[10] also studied the oncological safety of NSM in breast cancer treatment using data from the SEER database. However, their patient follow-up was relatively short. Only one- (100%) and 3-year CSS (97%) were reported. In our study population (N = 763), the 5- and 10-yr OS rates were 94.1% and 88.0% after a median follow-up of 69 months, respectively, suggesting the oncological safety of NSM in clinical practice. The CSS and OS of patients receiving NSM were, as expected, higher than those with non-NSM (total mastectomy or modified radical mastectomy) (S2 File). Selection bias plays a role in surgical decision making and patients who underwent a NSM likely had more favorable tumor characteristics. Similar OS (88–100%) of NSM have been observed in a series of retrospective studies[5,11–13] with follow-ups > 5 years. There have been two studies[14,15] from European countries that have reported survival rates of less than 80% with similar follow-up durations. It is possible that the patients included in these two studies were diagnosed and treated between 1988 and 2000 and before the development of the concept of the “molecular subtype of breast cancer”[16] and respective treatment strategies. Cruz et al [17] conducted a systematic review and meta-analysis including 20 studies with 5594 patients. They compared the DFS and OS following NSM and modified radical mastectomy/skin sparing mastectomy and did not show any significant differences between the procedures. Taken together, our study was consistent with others and confirmed the long-term oncological safety of NSM in breast cancer patients.

Although these retrospective data support the oncological safety of NSM, we should be aware that patients were carefully selected for inclusion in these studies, with favorable biological features of the tumor (lower tumor grade, negative nodes, no lymphovascular invasion, etc.), as suggested by the NCCN guidelines[7]. However, it is still unknown what specific criteria defines patient eligibility or ineligibility for NSM. In this study, there were 26%, 20% and 13.8% of the NSM cases with T2-3, N1-3 and ER negative disease, respectively. Whether the clinical outcomes associated with NSM were due to its use in these patients is a major concern. In this study, the 10-year OS was 72% for N2-3 patients after NSM. This was significantly better than the reported outcomes following traditional mastectomy in patients with a similar tumor burden. In our previous study[18], we obtained data from the National Cancer Database and showed that the 8-year OS rates were 66.6% and 53.5% following traditional mastectomy in N2-3 patients with and without RT, respectively. Additionally, the 10-year OS rate for N1 patients in the current study was 72.7%; this result was comparable with the findings of a study using data from the Netherlands cancer registry[19], which showed that the 10-year OS rate ranged from 65 to 75% in T1-2N1 patients with a traditional mastectomy (with or without RT). Therefore, we suggest that tumor size (T2 vs. T1) or nodal status (N2-3, N1 vs. N0) may not be contraindications for NSM in breast cancer patients.

A lack of information regarding the rate of NAC positivity and local recurrence was one of the limitations of our study. The potential that malignant tumor cells remain in the preserved nipple-areolar complex is a major concern regarding the oncological safety of NSM. Frozen section evaluation has been identified as an effective method to determine NAC status intraoperatively and thereby reduce the rate of NAC positivity. In a study by Eisenberg et al[20], 325 patients received a frozen section evaluation of their NAC. The sensitivity and specificity were determined to be 64% and 99%, respectively. Alperovich et al[21]. performed a frozen section evaluation of the NAC of 307 cases receiving NSM and reported a sensitivity and specificity of 58% and 100%, respectively. Therefore, with the use of frozen section evaluation, half of cases with positive NAC margins can be identified intraoperatively. Even in institutions without frozen section evaluation, the rate of NAC positivity has been identified as 2.7–14%[6,20,22]. Re-excision or observation alone in these patients seems to be safe, although follow-up has been relatively short[6,22]. Petit et al.[23] reported that when combined with intraoperative radiotherapy, patients with a positive NAC margin identified during NSM may not need further treatment after surgery. Taken together, preserving the NAC during NSM might not compromise the local control of breast cancer in these patients. This was also confirmed in a meta-analysis reported by Cruz et al [17], which showed that the local recurrence risk was 0–11.7% in studies with longer than 5 years follow-up.

Post-mastectomy RT (PMRT) has been identified as an important adjuvant therapy for local control after surgery. The NCCN has clear recommendations regarding the indications for PMRT based on tumor size and nodal and margin status[7]. However, real-world clinical practices in which more factors may be taken into consideration may not always follows these guidelines. For example, concerns related to leaving remnants of ductal tissue with the NAC may, theoretically, be more likely to prompt surgeons to recommend RT for NSM patients. Frasier et al.[24] used the SEER database and reported that PMRT was delivered in 29.9% and 7.4% of the patients in which RT was and was not recommended by the NCCN guidelines, respectively. Most of the reported studies[12,14,15,25] have shown that the rate of RT in NSM patients ranged between 15 and 30%. However, most of these studies were retrospective, used patients from a single institution, and did not provide detailed descriptions of the indications for RT. Hence, it was unclear whether RT was more likely to be delivered in these patients. In our study, the rates of RT were 61.4% (54/88) and 10.6% (201/1853) in the “RT recommended” and “RT not recommended” cohorts, respectively, which were higher than the rate of RT identified in Frasier’s study[24]. Our results were consistent with Agarwal’s study[26] and showed that NSM patients were more likely to receive RT than traditional mastectomy patients. However, whether PMRT in NSM patients is associated with improved local control and long-term survival remains unclear. There has been only one study[14] that reported a significant difference in local or regional recurrence depending on the use of RT (RT: 8.5%, No RT: 28.4%). However, several important factors, including tumor stage, were not adjusted for in that study’s model. Additionally, a plate of gland tissue (5 mm) with a 2 cm diameter was left around the NAC in their study, leading to a potentially higher risk of local or regional recurrences and, therefore, a potentially higher benefit of RT regarding local control. In our study, we did not observe any benefit in CSS to be associated with RT, suggesting that RT can be potentially avoided in more patients after NSM.

Several limitations of the SEER database should be noticed for accurate interpretation of the results of out study. First, the SEER database did not have information about systemic therapies, and therefore, their association with CSS in NSM patients cannot be assessed. Second, some important clinicopathological features were not available in the SEER database, such as tumor distance to nipple, Her2 status, margin status and lymphovascular invasion. Unable to adjust these factors may have influences on the analysis of risk factors of CSS. Third, inappropriate coding, suach as the underascertainiment of the RT receipt[27], may exist in the SEER database, which might bring bias to the study.

### Summary

The use of NSM has been increasing, and these data suggest that this procedure is oncologically safe in breast cancer patients, even in the presence of a high tumor burden. The use of RT after NSM identified in this study was also higher than that previously reported after traditional mastectomy. RT after NSM was associated with no benefit in terms of long-term survival. The role of RT after NSM in terms of local control remains unknown. We suggest that the indications for PMRT after traditional mastectomy may be used as reliable references for decision-making regarding the use of RT after NSM in breast cancer patients.

## Supporting information

S1 Fig Searched publications from Pubmed using “Nipple sparing mastectomy breast cancer” as terms in all fields. (PNG)

S1 File Coding for patient selection in the SEER database. (DOCX)

S2 File Comparison of CSS and OS in patients receiving NSM and non-NSM using the SEER database. (DOCX)
