# Supplementary figures and images for: Nipple sparing mastectomy in breast cancer patients and long-term survival outcomes: An analysis of the SEER database
Source: PLoS One. 2017 Aug 25;12(8):e0183448. doi: 10.1371/journal.pone.0183448 (PMC5571910; doi:10.1371/journal.pone.0183448)

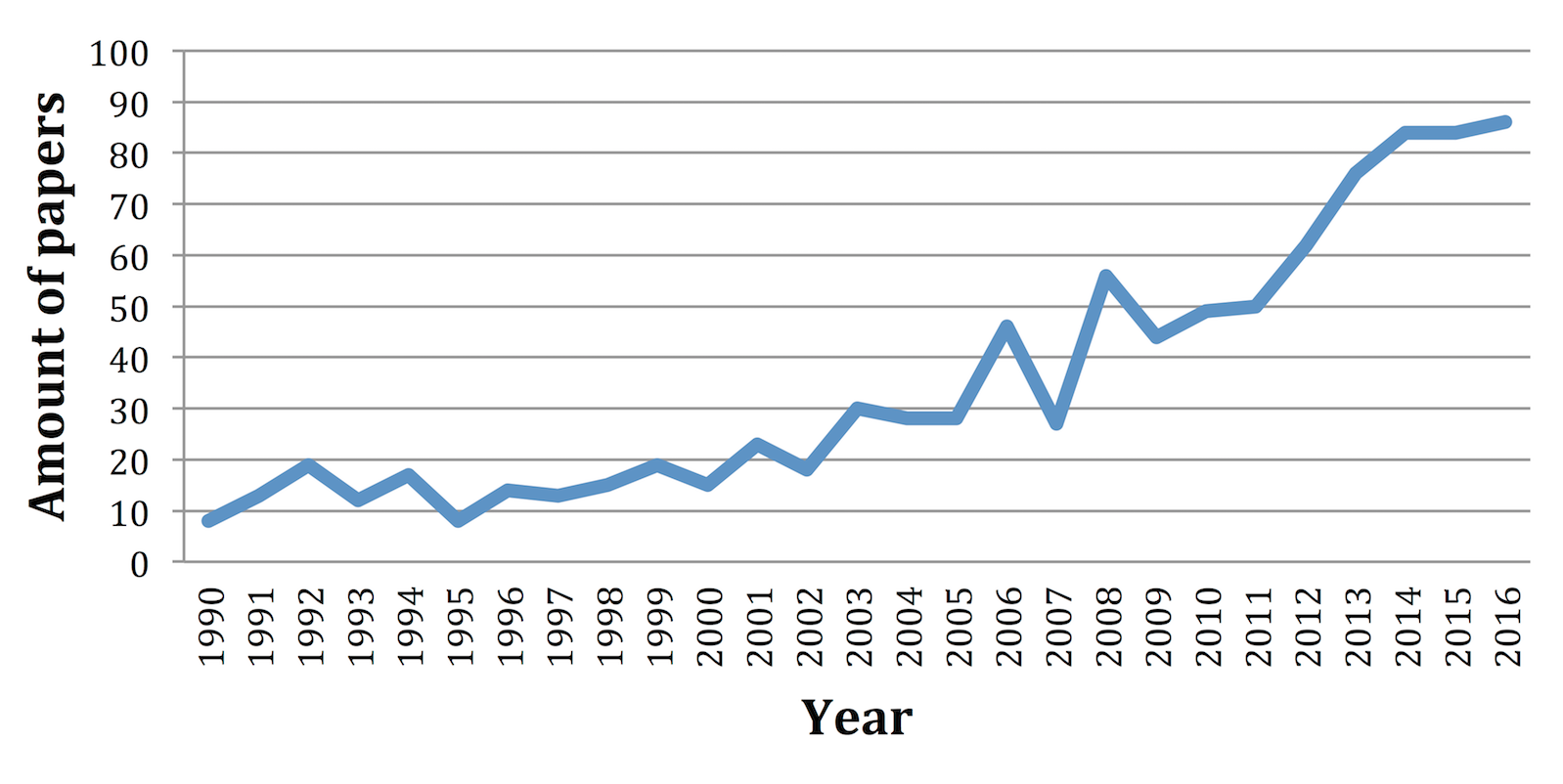

Supplement: S1 Fig — (PNG) [file pone.0183448.s001.png]
