# Supplementary material for: Nipple sparing mastectomy in breast cancer patients and long-term survival outcomes: An analysis of the SEER database
Source: PLoS One. 2017 Aug 25;12(8):e0183448. doi: 10.1371/journal.pone.0183448 (PMC5571910; doi:10.1371/journal.pone.0183448)
Supplement: S1 File — (DOCX) [file pone.0183448.s002.docx]

S1 File

Coding for patient selection in the SEER database.

{Race, Sex, Year Dx, Registry, County.Sex} = ' Female'

AND {Site and Morphology.Site recode ICD-O-3/WHO 2008} = ' Breast'

AND {Race, Sex, Year Dx, Registry, County.Year of diagnosis} = '1998','1999','2000','2001','2002','2003','2004','2005','2006','2007','2008','2009','2010','2011','2012','2013'

AND {Site and Morphology.Laterality} = 'Right - origin of primary','Left - origin of primary'

AND {Therapy.RX Summ--Surg Prim Site (1998+)} = 30

AND {Multiple Primary Fields.Sequence number} = 'One primary only','1st of 2 or more primaries'

AND {Cause of Death (COD) and Follow-up.Survival months} != 'Unknown'

AND {Stage - TNM.Breast - Adjusted AJCC 6th T (1988+)} = 'Tis','T1mic','T1a','T1b','T1c','T2','T3'
