# Supplementary material for: Nipple sparing mastectomy in breast cancer patients and long-term survival outcomes: An analysis of the SEER database
Source: PLoS One. 2017 Aug 25;12(8):e0183448. doi: 10.1371/journal.pone.0183448 (PMC5571910; doi:10.1371/journal.pone.0183448)
Supplement: S2 File — (DOCX) [file pone.0183448.s003.docx]

S2 File

To compare the survival of patients receiving NSM and non-NSM using the SEER database, we used similar data extract codes as described in S1 File, and the surgery code 40-42 (Total Mastectomy) and 50-52 (Modified Radical Mastectomy) to retrieve patients that had received non-NSM. A total of 205,896 patients were retrieved. Similarly, only patients diagnosed between 1998-2010 (N=169,292) were included for the survival analysis.

Comparison of CSS and OS in patients receiving NSM and non-NSM using the SEER database.

| Surgery | CSS | | | OS | | |
| --- | --- | --- | --- | --- | --- | --- |
|  | 5-year | 10-year | P | 5-year | 10-year | P |
| NSM | 96.90% | 94.90% | <0.01*; 0.015** | 94.10% | 88.00% | <0.01*; <0.01** |
| non-NSM | 90.60% | 83.80% |  | 82.30% | 67.10% |  |

* We used Kaplan-Meier Survival Analysis for univariate analysis.

**We used Cox regression and the T-stage, N-stage, age and tumor grade were adjusted.

NSM, nipple sparing mastectomy; CSS, Cancer Specific Survival; OS; Overall Survival;
